# Effects of unilateral real-time biofeedback on propulsive forces during gait

**DOI:** 10.1186/s12984-017-0252-z

**Published:** 2017-06-06

**Authors:** Christopher Schenck, Trisha M. Kesar

**Affiliations:** 1 Department of Biomedical Engineering, Georgia Institute of Technology, Atlanta, GA USA; 2 Division of Physical Therapy, Department of Rehabilitation Medicine, Emory University, 1441 Clifton Rd NE, Atlanta, GA 30322 USA

**Keywords:** Gait training, Real-time biofeedback, Ground reaction forces, Unilateral, Motor learning, Retention, Propulsion

## Abstract

**Background:**

In individuals with post-stroke hemiparesis, reduced push-off force generation in the paretic leg negatively impacts walking function. Gait training interventions that increase paretic push-off can improve walking function in individuals with neurologic impairment. During normal locomotion, push-off forces are modulated with variations in gait speed and slope. However, it is unknown whether able-bodied individuals can selectively modulate push-off forces from one leg in response to biofeedback. Here, in a group of young, neurologically-unimpaired individuals, we determined the effects of a real-time visual and auditory biofeedback gait training paradigm aimed at unilaterally increasing anteriorly-directed ground reaction force (AGRF) in the targeted leg.

**Methods:**

Ground reaction force data during were collected from 7 able-bodied individuals as they walked at a self-selected pace on a dual-belt treadmill instrumented with force platforms. During 11-min of gait training, study participants were provided real-time AGRF biofeedback encouraging a 20–30% increase in peak AGRF generated by their right (targeted) leg compared to their baseline (pre-training) AGRF. AGRF data were collected before, during, and after the biofeedback training period, as well as during two retention tests performed without biofeedback and after standing breaks.

**Results:**

Compared to AGRFs generated during the pre-training gait trials, participants demonstrated a significantly greater AGRF in the targeted leg during and immediately after training, indicating that biofeedback training was successful at inducing increased AGRF production in the targeted leg. Additionally, participants continued to demonstrate greater AGRF production in the targeted leg after two standing breaks, showing short-term recall of the gait pattern learned during the biofeedback training. No significant effects of training were observed on the AGRF in the non-targeted limb, showing the specificity of the effects of biofeedback toward the targeted limb.

**Conclusions:**

These results demonstrate the short-term effects of using unilateral AGRF biofeedback to target propulsion in a specific leg, which may have utility as a training tool for individuals with gait deficits such as post-stroke hemiparesis. Future studies are needed to investigate the effects of real-time AGRF biofeedback as a gait training tool in neurologically-impaired individuals.

**Electronic supplementary material:**

The online version of this article (doi:10.1186/s12984-017-0252-z) contains supplementary material, which is available to authorized users.

## Background

Individuals with post-stroke hemiparesis demonstrate reduced push-off force generation in the paretic leg during terminal stance phase, which can negatively impact gait speed, inter-limb symmetry, and walking function [1–5]. Restoration of normal push-off force generation is the focus of gait rehabilitation treatments such as fast treadmill walking [6–8] and functional electrical stimulation [9–11]. Push-off forces can be quantified by measuring anteriorly-directed ground reaction forces (AGRF) recorded from a force platform [1, 12]. Here, we test a unilateral AGRF biofeedback gait training tool that has potential for application in individuals with unilateral gait deficits such as post-stroke hemiparesis.

Real-time gait biofeedback is a potent tool that can enhance patient awareness of the impairment targeted during a gait retraining session, enabling self-correction of aberrant gait patterns [13]. Although there is a paucity of systematic investigations on the use of real-time biofeedback during post-stroke gait training, biofeedback during gait has been shown to be effective at modulating step asymmetry in post-stroke individuals [14, 15], trunk lean in able-bodied individuals [16], and knee adduction moment in individuals with varus knee alignment [17]. Force platform biofeedback during gait has been studied for improving limb loading symmetry following total hip arthroplasty [18] and gait asymmetry in trans-tibial amputees [19]. Previous investigations also used limb load monitors inserted within shoes to provide audio feedback about stance duration asymmetry post-stroke, but these monitors were not reliable for measuring GRFs [20, 21]. Recently, the advent and increasing popularity of instrumented treadmills has made it more feasible and convenient to provide real-time AGRF feedback during treadmill walking.

Real-time AGRF biofeedback was recently shown to be effective at increasing propulsion bilaterally in older adults [22]. For AGRF feedback to have utility in the rehabilitation of individuals with unilateral gait deficits, it would be beneficial to target propulsion symmetry, i.e., specifically target the reduced AGRF in the affected leg without proportionally increasing AGRF in the contralateral leg. During normal locomotion, push-off is known to modulate with gait speed and slope [23–27]. Previous investigations on able-bodied individuals used verbal instruction to increase push-off bilaterally [28, 29]. However, it is not known whether AGRF feedback can induce increases in propulsion unilaterally in one limb, instead of bilateral improvements in push-off force generation. We posit that the feasibility of using AGRF biofeedback to specifically target propulsion in the targeted leg should be demonstrated before AGRF biofeedback is used in the rehabilitation of individuals with unilateral gait deficits such as post-stroke hemiparesis. The objective of this study, therefore, was to investigate the use of AGRF biofeedback to unilaterally modulate propulsion during walking in able-bodied individuals. We hypothesized that a single session of unilateral AGRF biofeedback would lead to increased AGRF production in the targeted leg without increasing AGRF production in the contralateral leg.

## Methods

Seven young able-bodied individuals (age 27 ± 2 years, 1 male) participated in one session comprising treadmill walking at a self-selected speed. Participants were excluded if they had musculoskeletal or neurological disorders affecting gait. All participants reported their right leg as dominant. All participants provided informed consent and the study was approved by the Institutional Human Subjects Review Board.

### Determination of gait speed and targeted AGRF for biofeedback training

**Fig. 1:**
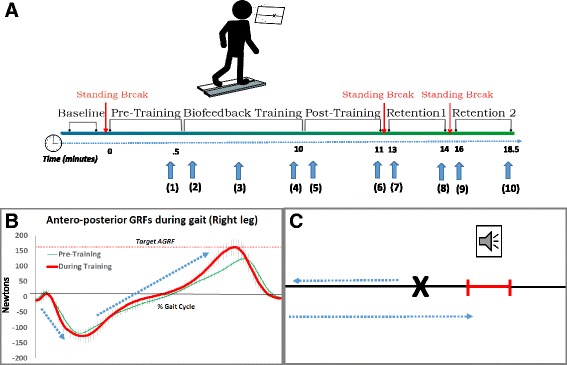
**a** Schematic of the experimental protocol. The participants walked on an instrumented treadmill and were provided visual and auditory feedback to increase peak AGRF in the right leg. The timeline of the experiment is shown at the bottom. The ten time points when AGRF data were collected from the right and left legs during the experiment are shown by vertical arrows. **b** Antero-posterior GRFs normalized to the gait cycle for a representative participant at baseline (*thin line*) and during biofeedback training (*bold line*). During biofeedback, the participant was able to increase the anteriorly-directed GRFs (positive forces in this graph) to match the target (shown by dashed horizontal line). *Error bars* show the participant’s standard deviation across 10 gait cycles. Additionally, *arrows* show the raw force data that match the biofeedback visual display arrow’s movement, shown in panel (**c**), during walking. **c** Schematic showing the biofeedback paradigm. The *black arrow* indicates the current antero-posterior GRF being generated during the stride cycle, and the bar indicates the targeted AGRF value. Visual feedback provides participants an estimate of how close or far they are from the targeted AGRF. An auditory tone provides auditory feedback indicating successful achievement of targeted AGRFs during the step cycle

At the beginning of the session, during baseline tests, the self-selected speed was determined for each participant by incrementally increasing treadmill speed by 0.1 m/s until the participants reported that the treadmill speed matched their comfortable walking pace. All gait tests and gait training were performed at this self-selected speed. Participants walked on a dual-belt instrumented treadmill with force platforms embedded under each belt (Bertec Corporation, Ohio, USA) with one foot on each treadmill belt to enable collection of GRF data from each leg. Next, baseline AGRF data were collected as the participant completed a 15-s walking trial, which were used to calculate the average peak AGRF produced by the right leg over 10 gait cycles. Using this baseline AGRF value, AGRF targets to be used during biofeedback were calculated as 20–30% greater than baseline (Fig. 1a). In the current study, we used peak AGRF to capture propulsive force generation because the feedback variable needed to be calculated accurately and rapidly. Peak AGRF was selected based on our pilot work showing that calculation of peak AGRF was more robust and less prone to error (e.g. due to errors in real-time gait event detection) compared to the AGRF integral. Additionally, a recent study [12] compared different AGRF metrics (impulse, peak, average) and showed that peak AGRF showed the strongest association with gait speed [12].

### Methodology for real-time biofeedback

During training, auditory and visual biofeedback was provided using a visual display screen and a speaker pointed toward the participant. The visual feedback display comprised a horizontal line graph with a moveable cursor that represented the current measured value of antero-posterior ground reaction force for the right leg (The MotionMonitor, Innovative Sports Training Inc., Illinois, USA) (Fig. 1c, Additional file 1: video S1). The targeted peak AGRF range appeared as a target line with vertical bars on either end, representing a 6-Newton error-tolerance range centered around the target AGRF (20–30% was greater than the baseline). The auditory feedback comprised an audible “beep” produced every time the cursor entered the target range, i.e. the participant achieved the targeted peak GRF value during their gait cycle.



**Additional file 1: Video S1.** Demonstrating the real-time AGRF biofeedback training session. The participant is walking on a split-belt treadmill instrumented with force platforms embedded within each belt. Visual feedback about AGRF generated by the right leg is provided on a projector screen displayed in front of the treadmill (The MotionMonitor, Innovative Sports Training Inc., Illinois, USA). The participant’s antero-posterior GRF is translated to the rightward-leftward motion of a marker on the screen. The target AGRF is shown as a bar. When a participant increases peak AGRF from the targeted (right) leg and achieves the target AGRF, an audible beep indicates success. (MP4 38411 kb)


### Gait training with biofeedback

Gait training comprised 11-min of continuous treadmill walking at the self-selected speed (1.1 ± 0.1 m/s). The gait training period was selected to match the estimated duration of continuous treadmill walking that an individual with neurologic impairment (such as stroke or multiple sclerosis) would be able to complete. During the first 30-s, participants were instructed to walk normally and the baseline value of each participant’s peak AGRF was recorded (Pre-training). Training with real-time biofeedback was provided during the next 10.5 min. The visual and auditory biofeedback was designed to encourage participants to increase the peak AGRF of their right leg 20–30% above their baseline peak AGRF. The biofeedback was presented in a faded feedback dosing schedule [30], comprising four 2-min periods of audio-visual biofeedback, interspersed by periods of walking without biofeedback. Periods of walking without biofeedback initially lasted 15 s and were subsequently increased stepwise by 15-s increments. Even during intervening training periods without biofeedback, participants were instructed to continue to make modifications to their gait that they used to achieve the targeted AGRF during periods with biofeedback. After completion of 10.5 min of biofeedback training, two 30-s post-training trials were collected during walking without biofeedback (Post1 and Post2). Comparisons of peak AGRF generated by the right leg during post-training versus pre-training were used to evaluate whether gait training with AGRF biofeedback produced immediate improvements in peak AGRF (acquisition of a new gait pattern).

### Retention tests following gait training

After the 11-min of biofeedback gait training, participants were provided a 2-minute standing break. The treadmill was then moved to the self-selected speed again and participants were instructed to walk normally for 30 s (Retention 1 Normal Gait). Next, participants were instructed to modify their gait to match the walking pattern they remembered employing during the biofeedback training (Retention 1 Replicate Gait). Peak AGRF values collected during these retention test trials were used to determine whether the participants could demonstrate recall of the gait pattern learned during biofeedback training (Replicate Gait), and to assess whether they could switch between the newly-learned gait pattern and their ‘normal’ gait pattern upon instruction (Normal Gait). After another 2-min standing break, similar procedures were performed (normal and replicate gait pattern) during a 2nd retention test comprising 2-min of walking without biofeedback. The 2-min breaks between the retention tests were utilized to determine whether the passage of time (as well as intervening walking tests) would lead to a losses in AGRF gained during the training period.

#### Data analysis and dependent variables

##### Primary dependent variables

GRF data averaged across 10 consecutive gait cycles were used to evaluate the peak AGRF during each period of the experiment. Data collected during pre-training, early (first 30-s), mid- (during the 5th minute of training) and late (during the 10th minute of training) periods of training, and post-training provided peak AGRF values before, during, and immediately after training. Additionally, GRF data from the retention tests helped to evaluate whether increased peak AGRF generation for the targeted (right) limb persisted following standing rest breaks, as well as following brief periods of ‘normal’ ambulation without focus of attention on AGRF production. Our primary dependent variable was the peak AGRF from the targeted (right) leg.

Additional outcome variables included the peak AGRF for the non-targeted (left) leg, coefficient of variance of peak AGRF for both targeted and non-targeted legs, as well as error between targeted and generated peak AGRFs for the right leg. Coefficient of variance (CV) in peak AGRF production was calculated to examine whether step-to-step variability in AGRF production changed over time during training. Percent error was computed as the difference between the targeted versus actually generated peak AGRF with respect to the targeted peak AGRF. Percent error in peak AGRF production in the targeted leg was compared to assess whether participants had a tendency to undershoot or overshoot with respect to the targeted AGRF. Root mean square error (RMSE) in peak AGRF, calculated as the square root of the mean sum of squared differences between target and measured peak AGRF, was also compared to evaluate magnitude of error between the targeted and actual AGRFs.

##### Secondary dependent variables

Additional gait variables were calculated for both the targeted (right) and non-targeted (left) limb, included the timing of peak AGRF generation with respect to the stance phase, cadence (steps per minute), temporal parameters (stride duration, stance duration, swing duration), and peak vertical GRF. Gait events (heel strike and toe off) were identified bilaterally in Motion Monitor software (Innovative Sports Training Inc., Illinois, USA). Stance duration was computed as the time between heel strike and the subsequent toe off for the same leg. Swing duration was computed as the time between a toe off and the subsequent heel strike for the same leg. Peak vertical GRF was calculated as the maximum VGRF value during the stance phase. Timing of peak AGRF was detected as the time from the previous heel strike to the time point at which the maximum AGRF was detected, and normalized to stance phase duration. The secondary gait variables were evaluated at 3 time points – Pre-training, Post1, and Retention 2-Replicate Gait.

##### Statistical analysis

One-way repeated measures analysis of variance (ANOVA) were performed to determine the effect of time during training on each of the primary dependent variables. The 10 time points included in the ANOVA were: (1) Pre-training; (2–4) Early, Mid, and Late training - during the 1st, 5th, and 10th minute of training, respectively, with biofeedback present; (5–6) Post1 and Post2 - first and last 10 gait cycles collected during 60-s post-test without biofeedback immediately following training; (7) Retention 1-Normal Gait, (8) Retention 1-Replicate Gait; (9) Retention 2-Normal Gait; and (10) Retention 2-Replicate Gait (Fig. 1a). If the ANOVA showed a significant main effect, post hoc pairwise comparisons were performed using the Bonferroni correction for multiple comparisons to compare pre-training values with each of the other time points. For the secondary gait variables, paired t-tests (with Bonferroni correction) were performed to compare data at Pre-Training with Post1, and Pre-Training with Retention 2-Replicate Gait. Significance level was set at α ≤ 0.05 for all tests.

## Results

### Peak a-GRF production

**Fig. 2:**
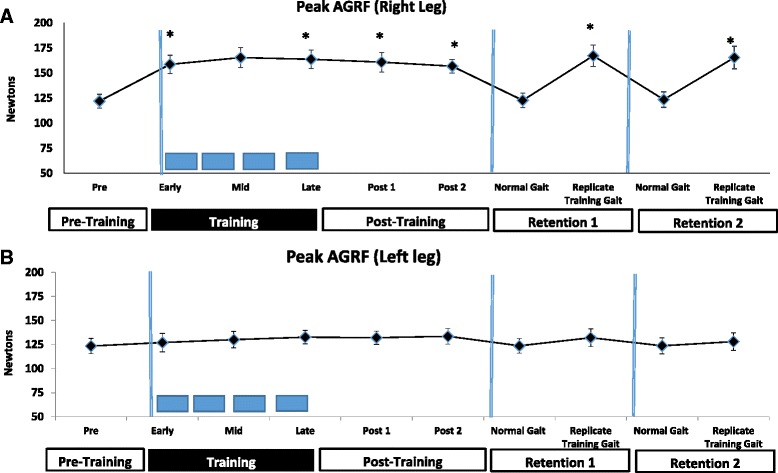
**a** Peak AGRF (N=7, error bars indicate standard error) for the targeted (*right*) leg before, during, and after biofeedback training. During biofeedback training period, the approximate durations when feedback was on versus off (faded feedback schedule, described in the methods) are indicated by colored bars along the x-axis. Standing breaks are indicated by vertical lines intersecting the x-axis. * indicate significantly greater peak AGRF compared to pre-training (*p*<0.05). **b** Peak AGRFs for the non-targeted or left leg did not show change during training

Complete data were collected on all 7 participants. Representative anterior-posterior GRF data from one of the participants (normalized to the gait cycle) showed increases in anteriorly-directed GRFs for the targeted (right) leg during the propulsive phase of the gait cycle (terminal stance phase) during biofeedback training compared to pre-training (Fig. 1b). The one-way ANOVA assessing overall effect of time on peak AGRF of the limb targeted during training (right leg) showed a significant main effect of time (F = 4.67, *p*=.001). Planned post hoc comparisons with Bonferroni correction for multiple comparisons between pre-training and each of the other time points revealed a significant difference between the pre-test versus early-training (*p*=.008), mid-training (*p*=.017), late training (*p*=.001), Post1 (*p*=.005), Post2 (*p*=.027), Retention1-Replicate Gait (*p*=.012), and Retention2-Replicate Gait (p=.048) (Fig. 2a). There was no difference in peak AGRF production between Pre-training and Retention1-Normal Gait or Retention2-Normal Gait (p>.9). The one-way ANOVA for the non-targeted (left) leg showed no significant effect of biofeedback over time on peak AGRF production (*p*=0.068, F=1.926) (Fig. 2b).

### Coefficient of variance in peak AGRF during and after biofeedback training

**Fig. 3:**
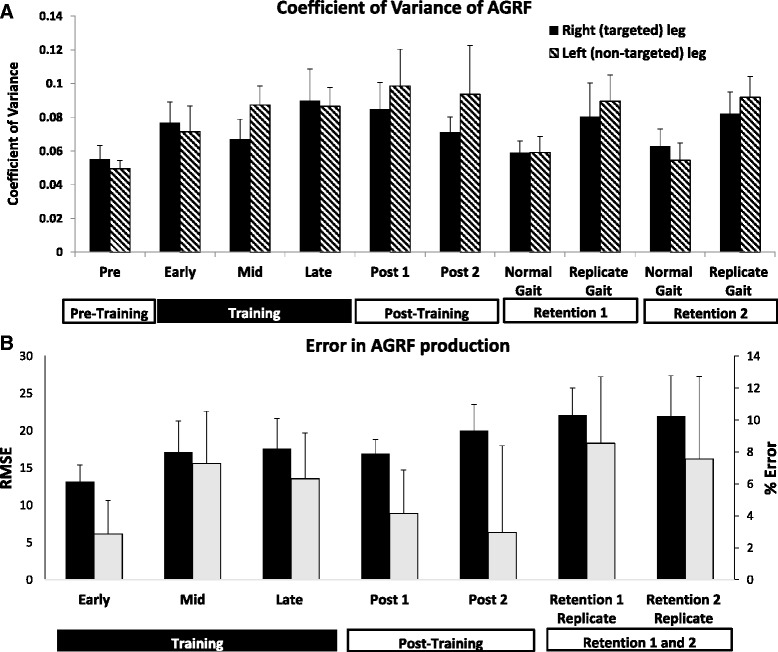
**a** Average (N=7) coefficients of variation (CV) for the peak AGRF generated by the right (*solid bars*) and left (*hatched bars*) legs during the different time periods within the experiment. CVs, a measure of stride-to-stride variability in peak AGRFs, were calculated for each participant using data for the 10 gait cycles that were used to determine the average peak AGRF for each time point during the experiment. **b** Average (N=7) RMSE (*black bars*) and percent error (*gray bars*) for peak AGRFs for the right leg during different time periods within the experiment

The one-way ANOVA showed no overall effect of biofeedback over time on CV of peak AGRF production in the targeted leg (*p*=0.453, F=.997) (Fig. 3a). Interestingly, the non-targeted leg showed a significant overall effect of time (*p* = 0.009, F=2.822), but none of the post-hoc pair-wise comparisons were significant when adjusted for multiple comparisons (all p’s>.74) (Fig. 3a). Although there was no statistical difference in the CV, the average CV increased from 0.06±.02 during pre-training to 0.09±.05 during late- training for the targeted leg (Fig. 3a). Similarly, the average CV for the non-targeted limb also increased from 0.05±.01 during pre-training to 0.09±.03 during late-training (Fig. 3a).

### Error in achieving targeted peak AGRF during and after biofeedback training

Individual participant data as well as group averages for % error showed a tendency to overshoot the target AGRF or positive % errors during training (Fig. 3b). The one-way ANOVA showed no significant effect of biofeedback over time on percent error in peak AGRF production (*p*=.690, F=.65) or RMSE in peak AGRF production (*p*=.448, F=.99) in the targeted leg (Fig. 3b).

### Secondary gait variables

#### Timing of peak AGRF

**Fig. 4:**
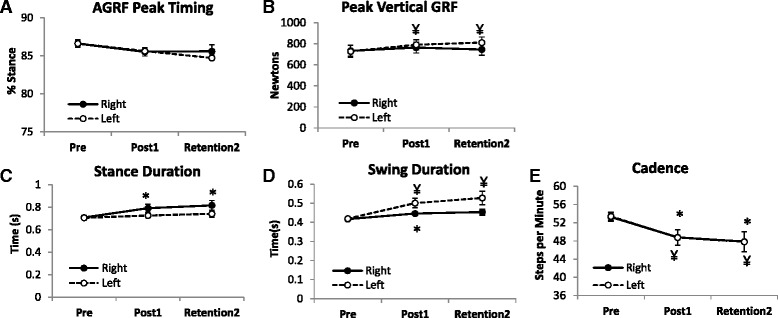
Average (N=7) values for secondary gait variables for the right (filled symbols) and left (unfilled symbols) legs during pre-training (Pre), immediately following training (Post1), and during the Retention2 Replicate gait test (Retention2). Graphs are shown for the timing of peak AGRF (**a**), peak vertical GRF (**b**), stance duration (**c**), swing duration (**d**), and cadence (**e**). * indicates that the *t*-test detected a significant difference compared to Pre-training (*p*≤0.05) for the right leg (limb targeted during biofeedback training). ¥ indicates that the *t*-test detected a significant difference compared to Pre-training (*p*≤0.05) for the left leg (non-targeted limb)

Compared to pre-training, the timing of peak AGRF generation (with respect to the stance phase duration) showed no difference at Post1 or Retention 2-Replicate Gait for the right or left leg (all p’s>0.1) (Fig. 4).

#### Vertical GRF

No significant differences were observed in the right peak vertical GRFs at Post1 (*p*=0.13) or retention2 (*p*=1.13) compared to pre-training. Left peak vertical GRF was significantly greater at Post1 (*p*=0.04) as well as at Retention 2 (*p*=0.03) compared to pre-training (Fig. 4).

#### Cadence

Compared to pre-training, cadence for the right (targeted) limb showed a reduction at Post1 (*p*=0.01) and Retention 2-Replicate Gait (*p*=0.02). Cadence for the left limb was significantly smaller at Post1 (*p*=0.01) and Retention2 (*p*=0.02) compared to pre-training (Fig. 4).

#### Temporal parameters

Right stance duration showed a significant increase at Post1 (*p*=0.02) and Retention2 (*p*=0.03) compared to pre-training (Fig. 4). Compared to pre-training, right swing duration increased at Post1 (p=0.05), and showed a statistical trend for an increase at Retention2 (*p*=0.09). Left stance duration showed no significant change at Post1 or Retention 2 (both p’s>0.31). Left swing duration (which is equivalent to the right single limb support time) showed a significant increase at Post1 (*p*=0.005) and Replicate 2 (*p*=0.03) compared to pre-training (Fig. 4).

## Discussion

Study participants demonstrated increased peak AGRF production in the targeted leg during and immediately after the 11-min bout of AGRF biofeedback training. Participants successfully increased peak AGRF production in the targeted leg within the first minute of the biofeedback-guided gait training (i.e. early training), and maintained the increased AGRF production throughout the remainder of the biofeedback training period (i.e. at mid- and late-training). Following withdrawal of biofeedback immediately after training, increased peak AGRFs continued to be demonstrated during post-tests (Post1 and Post2) performed in the absence of biofeedback. Demonstration of increased peak AGRF during the biofeedback training, and the persistence of the increased peak AGRF generation in the absence of biofeedback immediately following the training period suggest the acquisition of a modified gait pattern in response to the biofeedback. More importantly, during retention tests performed after standing rest breaks (retention 1 and retention 2 replicate trials), even in the absence of biofeedback, participants successfully generated increased peak AGRF in the targeted leg upon instruction to replicate gait modifications they remembered from the biofeedback training period. Compared to pre-training, participants demonstrated longer stance duration and reduced cadence in the targeted (limb) following biofeedback training. Increased peak AGRF production in the targeted leg was not demonstrated after training when participants were provided instructions to walk “normally”, indicating that participants were able to switch between their normal baseline pattern of AGRF generation, and the modified gait pattern associated with greater AGRF production acquired during the biofeedback training.

While the targeted (right) limb increased AGRF production during and after training, no change in peak AGRF was observed in the non-targeted (left) leg, suggesting that real-time gait biofeedback may target AGRF production unilaterally in a single leg without modulating peak AGRF generation in the contralateral non-targeted leg. In able-bodied individuals, total propulsion during the gait cycle is contributed equally by the right and left legs, leading to symmetrical peak AGRFs for both limbs. Greater peak AGRF generation by the targeted right leg without a concomitant increase in AGRF for the left leg suggests that biofeedback training induced an asymmetry in AGRF production in able-bodied individuals, with the right (targeted) leg contributing more to total propulsion than the left leg. This specificity of targeted increase in AGRF to the leg receiving biofeedback may be valuable as a treatment strategy for post-stroke individuals, who typically demonstrate reduced AGRF production in the paretic leg, and reduced contribution of the paretic limb to total propulsion [1].

Previously, Franz and colleagues demonstrated that older adults have an underutilized propulsive reserve by showing that older adults could increase push-off force generation compared to baseline when provided real-time biofeedback of AGRF [20]. The primary difference in methodology between our current study and the previous demonstration of real-time propulsive feedback is that Franz and colleagues targeted bilateral increases in propulsion. Our study demonstrates the specificity of a unilateral AGRF biofeedback paradigm in increasing AGRF in the targeted leg without causing significant change to AGRF in the non-targeted leg. Furthermore, unlike our study, Franz and colleagues studied the immediate effects of feedback provided for shorter durations (2-min), and did not test for the presence of retention following standing breaks to assess the recall or persistence of the gait patterns acquired during biofeedback.

The lack of significant changes in the coefficient of variance and error of peak AGRF production in the targeted leg over time was somewhat surprising because we hypothesized an increase in stride-to-stride CVs for AGRF as changes in gait performance occur during acquisition of a modified gait pattern. The accurate AGRF biofeedback provides knowledge of performance during training, potentially leading to greater stride-to-stride consistency in peak AGRFs. Perhaps able-bodied individuals have sufficient robustness and redundancy in their motor control to rapidly increase AGRF production without a marked increase in stride-to-stride variability during gait pattern modification. Similarly, with regards to percent error and root mean square error in peak AGRF, contrary to what we expected, participants did not demonstrate greater errors during the early training period, and a reduction of error as training progressed. Again, the lack of difference in peak AGRF error in able-bodied individuals may showcase the speed and ease with which the able-bodied locomotor control circuitry can adapt and acquire a new gait pattern. In a previous investigation on the biomechanical mechanisms underlying propulsion during gait, able-bodied individuals increased their push-off forces by ~27% in response to verbal instruction to walk with greater ankle push-off, providing evidence for the adaptability of locomotion [28, 29, 31].

We also investigated whether AGRF biofeedback training induced changes in other spatio-temporal gait parameters. Following the AGRF feedback training period, study participants demonstrated longer stance duration, single limb stance duration, and swing duration in the targeted (right) limb. Because the participants’ speed was determined by the treadmill belts, the increase in peak AGRF was accompanied by a reduction in cadence. Increased peak AGRF was also accompanied by a greater peak vertical GRF force generation on the contralateral (left) limb, likely caused by the redistribution of weight during push-off force generation (terminal stance phase) from the right leg. Although there was a prolongation of stance duration following training, there was no change in the timing of occurrence of peak AGRF.

Based on previous studies using correlations to determine the factors that contribute to propulsive force generation during gait, we hypothesize that the biomechanical strategies used by the participants to increase AGRF production in the targeted leg during gait biofeedback training may include increased step length, trailing limb angle, and ankle plantar flexion moment in the targeted (right) limb [2, 5, 10, 32–34]. Our study was, however, limited by the lack of kinematic data. Future studies are needed to elucidate changes in ankle, knee, and hip kinematics that contribute to increased peak AGRF. Because we do not provide participants with specific verbal instructions, cues, or biofeedback about other aspects of their gait except peak AGRF, we postulate that real-time biofeedback-guided training offers the participant the ability to experiment with different biomechanical strategies to achieve the desired modulation of the targeted gait parameter, such as peak AGRF production. Our present study findings suggest that the use of real-time gait AGRF biofeedback as a gait training tool in clinical populations such as stroke merits further investigation. As a gait training intervention, AGRF biofeedback may empower an individual to experiment with different biomechanical strategies customized to their own clinical impairments to achieve the targeted increase in peak AGRF. In the current study, all participants achieved the task of increasing peak AGRF production with low to moderate difficulty. In terms of feasibility, two participants reported slight joint discomfort in the right hip or knee immediately following training; however, no one experienced long-lasting discomfort.

The current study demonstrates the short-term effects of AGRF-guided gait biofeedback as a gait training tool to unilaterally target propulsion. Ongoing studies in our laboratory are exploring AGRF biofeedback as a stroke gait retraining strategy. We posit that the ability of AGRF-guided biofeedback to target only the paretic leg may offer an advantage over existing stroke rehabilitation interventions such as treadmill training, over ground training, and body-weight supported training, which involve bilateral practice without specifically targeting the paretic leg. Additionally, knowledge of performance provided by the AGRF-guided biofeedback may enhance the patient’s engagement during training, and increase the efficacy of rehabilitation. Similar to previous investigations employing biofeedback as a post-stroke gait training tool for targeting spatio-temporal parameters such as step length and stance time [14, 15], we believe that AGRF-based feedback may be a valuable gait training strategy worthy of investigation. Reduced AGRF has been demonstrated to be an important biomechanical impairment in people post-stroke, and has been associated with over ground gait speed, gait asymmetry, and stroke severity [1, 2, 12, 35]. Furthermore, there may be differences in motor learning processes associated with feedback based on force-generation (such as AGRF) versus kinematic or spatio-temporal variables (step length or stance time), justifying the need to specifically evaluate AGRF biofeedback. The feedback-parameter employed during gait training (e.g. peak AGRF, stance time, step length) can also be customized to an individual’s gait impairments.

Future studies are needed to determine if unilateral AGRF biofeedback is effective at increasing AGRF production in the paretic leg of individuals with unilateral gait deficits such as post-stroke hemiparesis, and to determine whether real-time AGRF biofeedback training leads to improvements in other gait kinematic and kinetic biomechanical parameters. Additional investigations may also be needed to determine how the training structure, schedule, dosage or magnitude of targeted peak AGRF (30% versus 50% greater than baseline AGRF), and type of biofeedback (e.g. auditory, visual, or combined auditory and visual feedback) influence motor learning during gait training.

## Conclusions

We demonstrated the feasibility of real-time unilateral AGRF biofeedback as a gait training approach, and showed that able-bodied individuals demonstrated short-term recall of modified gait patterns following a short period of AGRF biofeedback training. This study lays the groundwork for testing the feasibility of similar AGRF biofeedback intervention strategies in clinical populations with unilateral gait deficits such as individuals post-stroke.

## Abbreviations

AGRF: Anterior ground reaction force
ANOVA: Analysis of variance
CV: Coefficient of variation
GRF: Ground reaction force
RMSE: Root mean square error
